# Non-Surgical Repair of Internal Resorption with MTA: A Case Report

**Published:** 2012-10-13

**Authors:** Zahed Mohammadi, Mohammad Yazdizadeh, Sousan Shalavi

**Affiliations:** 1. Department of Endodontics, Hamedan University of Medical Sciences, Hamedan, Iran; 2. Department of Endodontics, Ahvaz University of Medical Sciences, Ahvaz, Iran; 3. Hamedan University of Medical Sciences, Hamedan, Iran

**Keywords:** Mineral Trioxide Aggregate, Perforation, Root Resorption

## Abstract

Internal resorption is rare in permanent teeth. Treatment is usually performed through warm gutta-percha technique. If the resorptive process perforates the root, treatment may be more difficult and is usually performed via surgical approach. Non-surgical repair of a perforating internal root resorption with MTA was conducted in this case. Before repairing the resorption, a master gutta-percha point was placed in the canal to maintain negotiability of the original canal path. Then, MTA was prepared and applied with a small carrier in the resorption area and compacted. Thereafter gutta-percha was retrieved and the access cavity was closed with a temporary filling material. In the second visit, the root canal was obturated with gutta-percha and AH26 sealer using lateral compaction technique and subsequently, the crown was restored. The symptoms and signs ceased and the result was satisfactory at the 18 month follow-up visit.

## Introduction

Internal resorption is a rare type of root resorption in permanent teeth; this pathological lesion is related to pulpal inflammation and bacterial invasion. The tooth may be restored or caries-free. The defect may be located anywhere within the root canal system. The usual radiographic presentation of internal root resorption is a fairly uniform radiolucent enlargement of the pulp canal. Because the resorption is initiated in the root canal, the resorptive defect includes some part of the root canal space. Therefore, the original outline of the root canal is distorted [1][2]. Only on rare occasions, when the internal resorptive defect penetrates the root and impacts the periodontal ligament, does the adjacent bone show radiographic changes. If untreated, the lesion will progress and will eventually perforate the wall of the root. The destruction of dentin may be so severe that the tooth fractures [1].

Like that of other inflammatory resorptive defects, the histologic picture of internal resorption is granulation tissue with multinucleated giant cells. An area of necrotic pulp is found coronal to the granulation tissue. Dentinal tubules, which contain microorganisms and communicate between the necrotic zone and the granulation tissue, can sometimes be seen. Unlike external root resorption, the adjacent bone is not affected with internal root resorption [2].

As the resorptive defect is the result of the inflamed pulp and the blood supply to the tissue is through the apical foramina, the correct approach to treatment is endodontic treatment that effectively removes the blood supply to the resorbing cells. After adequate anesthesia has been administered, the canal apical to the internal defect is explored and a working length short of the radiographic apex is used. The apical canal is thoroughly instrumented to ensure that the blood supply to the tissue resorbing the root is cut off. Once root canal instrumentation has been completed, it should be possible to obtain a blood free dry canal when the roots are checked with paper points. Calcium hydroxide is then spun into the canal to facilitate the removal of the tissue in the irregular defect at the next visit. At the second visit, the tooth and defect are filled using a soft gutta-percha technique [2].

MTA has been developed to seal off pathways of communication between the root canal system and the external surface of the tooth. It is marketed in grey-colored and white-colored preparations; both are 75% Portland cement, 20% bismuth oxide, and 5% gypsum by weight. MTA is a powder that consists of fine hydrophilic particles that forms a colloidal gel in the presence of water or moisture, which finally solidifies to form hard cement within approximately 3 hours [3]. The principal components of the MTA are tricalcium oxide, tricalcium aluminate, tetracalcium aluminoferrite, and calcium sulfate dehydrate [3][4]. The particle sizes ranges from 1-10 mm for gray MTA powder, the white MTA powder has particles less than 1 to approximately 30 mm before hydration [3]. The pH of MTA is 10.2 after mixing and rises to 12.5 after 3 hours [4].

It has a variety of potential uses such as vital pulp therapy, apexification, perforation repair, root-end filling, apical barrier and root canal obturation [5]. The purpose of this paper was to report repair a large internal resorptive defect using MTA.

## Case report

**Figure 1:**
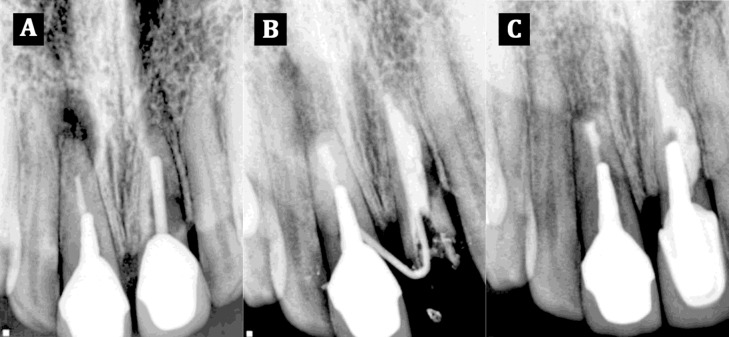
A) Preoperative radiograph; B) Root filling radiograph; C)Eighteen months follow-up radiograph

A 38-year old female suffered from a buccal swelling in the maxillary anterior region for several weeks. She was referred to a private endodontist practice. Her medical history was noncontributory and history of dental trauma was also denied. The patient also reported a history of previous root canal therapy of both maxillary central incisors two years ago. After taking a periapical radiograph an incomplete endodontic treatment and a large resorptive area was observed in the maxillary right central incisor (Figure 1A).

Furthermore, the depth of gingival crevice around this tooth was 3-4 mm. After administration of local anesthesia, the crown and the post inside the canal were removed with crown remover (Crown-A-Matic, Peerless International Inc., Stoughton, Mass, USA).

After cleaning and shaping of the root canal and irrigation with 1.3% sodium hypochlorite (Merck, Darmstadt, Germany), the canal was medicated for two weeks with calcium hydroxide (Golchai, Tehran, Iran) mixed to a creamy consistency. At the second visit calcium hydroxide was removed with master apical file and EDTA irrigation (Golchai, Tehran, Iran). Thereafter, in order to remove remnants of EDTA, root canal was completely irrigated with sterile normal saline. Before repairing the resorption, a master gutta-percha point was placed in the canal to maintain the negotiability of the original canal path. Afterwards, a thick mix of MTA (Angelus Industria de Productos Odontologicos S/A, Londrina, Brazil) was prepared and applied with a small carrier in the resorption area and compacted with a small moistened cotton pellet (Figure 1B). Thereafter, gutta-percha was retrieved, a moist cotton pellet was placed on the MTA and the access cavity was closed with a temporary filling material. The temporary filling material was removed after 24 hours and the setting of MTA was confirmed using an explorer. Finally, the root canal was obturated with gutta-percha and AH26 (Dentsply, Konstanz, Germany) sealer using lateral compaction technique and the crown of the tooth was restored with a post-core and crown (Figure 1C). The clinical follow-up at 18 months showed adequate clinical function and absence of clinical symptoms and sinus tract (Figure 1C).

## Discussion

Root perforations adversely affect the prognosis of teeth [6]. MTA is known as a biocompatible material that may induce cementum formation when applied to perforations and when used as a root end filling. Clinical reports of human subjects have also proved that MTA is suitable material for perforations. It can function in the presence of moisture and inhibits the activity of bacteria [3]. Treatment of non-perforated internal resorption is relatively easy and is usually performed through warm gutta-percha technique [1]. However, treating internal root resorption that has perforated is more difficult and is usually performed through surgical approach [1]. It should be noted that the internal (non-surgical) approach is preferred to seal the perforations [7].

Therefore, we decided to repair the resorption area through non-surgical approach. Few studies associated with MTA repair of perforating internal resorption could be found. Hsien et al. reported successful surgical repair of a large perforating internal root resorption over one year follow-up [8]. In another report, Sari and Sonmez reported successful MTA repair of a cervical root resorption of a mandibular second primary molar with 18 months follow-up period [9]. Meire and De Moore repaired a perforating internal resorption in the mesial root of a mandibular second molar and found that after 2 years no clinical abnormalities were found, and complete resolution of the alveolar bone lesion and establishment of a new periodontal ligament were observed [10]. In another case report, Takita et al. reported a maxillary right lateral incisor with a perforating internal resorption in a 50-year-old woman and found that 3 years after repairing the defect with MTA, the resorption area was adequately repaired and the tooth was asymptomatic [11].

Jacobovitz and de Lima reported a maxillary central incisor with perforating internal resorption; 20 months after repairing the resorption area with MTA patient was asymptomatic and functional [12].

Brito-Junior et al. also used MTA to manage non-surgical treatment of a case with perforating internal root resorption [13]. The clinical findings and periapical radiographs indicated success at the 2 year follow-up. However, after 8 years the radiograph showed an extensive radiolucent area in the middle third of the root with separation of the apical and coronal root segments. Other studies did not have such long follow ups; most reported favourable outcomes in shorter time periods. Using clinical and radiographic criteria, Silveira et al. reported the favourable response of internal resorption to MTA treatment after 18 month follow-up [14].

In a case report, the application of MTA to restore a perforating internal resorptive defect in maxillary left central incisor with sinus tract, induced osseous repair of the previous periapical pathosis at the one-year radiographic recall [15]. The patient was asymptomatic, with the tooth exhibiting normal probing, mobility, and function.

## Conclusions

In conclusion, it seems that MTA is an appropriate material to manage perforating internal root resorption and has shown success over 1-2 year follow-ups.
